# USAG-1/SOSTDC1 as a Candidate Target for Periodontal Regeneration: Biological Rationale, Knowledge Gaps, and Future Directions

**DOI:** 10.3390/molecules31193389

**Published:** 2026-09-23

**Authors:** Nada Tawfig Hashim, Bakri Gobara Gismalla, Ayman Ahmed, Muhammed Mustahsen Rahman, Vivek Padmanabhan, Md Sofiqul Islam, Riham Mohammed, Mariam Elsheikh, Shadi El Bahra

**Affiliations:** 1 Department of Periodontics, RAK College of Dental Sciences, RAK Medical & Health Sciences University, Ras-Al Khaimah 12973, United Arab Emirates; 2 Department of Oral Rehabilitation, Faculty of Dentistry, University of Khartoum, Khartoum 11115, Sudan; 3 Department of Periodontology and Implantology, College of Dentistry, The National Ribat University, Khartoum 11115, Sudan; 4 Department of Pediatric and Preventive Dentistry, RAK College of Dental Sciences, RAK Medical & Health Sciences University, Ras-Al Khaimah 12973, United Arab Emirates; 5 Department of Operative Dentistry, RAK College of Dental Sciences, RAK Medical & Health Sciences University, Ras-Al Khaimah 12973, United Arab Emirates; sofiqul.islam@rakmhsu.ac.ae; 6 Department of Oral Surgery, RAK College of Dental Sciences, RAK Medical & Health Sciences University, Ras-Al Khaimah 12973, United Arab Emirates; 7 Department of Oral &Maxillofacial Surgery, Faculty of Dentistry, University of Khartoum, Khartoum 11115, Sudan or m.elsheikh@ajman.ac.ae; 8 Clinical Sciences Department, College of Dentistry, Ajman University, Ajman P.O. Box 346, United Arab Emirates

**Keywords:** USAG-1, SOSTDC1, periodontal regeneration, BMP signalling, Wnt signalling, cementogenesis, alveolar bone, periodontal ligament stem cells, regenerative periodontology

## Abstract

Background: Uterine Sensitization-Associated Gene-1 (USAG-1), also known as SOSTDC1, is a bifunctional antagonist of bone morphogenetic protein (BMP) and Wnt signalling—two pathways that are central not only to tooth development but to the entire repertoire of periodontal tissue biology. Current periodontal regenerative therapy relies on exogenous BMP delivery and barrier membranes, approaches with significant limitations in predictability and tissue specificity. The recent entry of anti-USAG-1 antibody therapy into early-phase human clinical trials, with congenital tooth agenesis as the lead indication, raises the question of whether targeted USAG-1 inhibition could be repurposed for periodontal regeneration. Purpose: This perspective examines the biological rationale for USAG-1 as a target in periodontal regeneration, maps the plausible mechanisms through which its inhibition could restore cementum, periodontal ligament (PDL), and alveolar bone, and outlines the conditions and research agenda under which such an approach could be clinically developed. Conclusions: USAG-1 transcripts have been reported in adult gingival tissues, and USAG-1 antagonises BMP and Wnt signals that are implicated in cementogenesis, PDL fibre formation, and alveolar bone homeostasis. Its inhibition is proposed as a biologically plausible, hypothesis-generating strategy for amplifying endogenous regenerative signalling in the periodontium, which might differ from, and could potentially complement, existing regenerative protocols; neither possibility has yet been tested. Realising this potential will require localised delivery strategies, validated biomarker endpoints, and controlled trials embedded in the framework of the EFP S3 stepwise treatment algorithm. Direct experimental evidence that USAG-1 inhibition promotes periodontal regeneration is currently lacking; the case advanced here is inferential and hypothesis-generating, not a demonstrated therapeutic effect.

## 1. Introduction

Periodontitis results in progressive, irreversible destruction of the tooth-supporting apparatus: the cementum, periodontal ligament (PDL), and alveolar bone [1,2]. Despite decades of advances in regenerative periodontology—guided tissue regeneration, bone grafting, enamel matrix derivatives, recombinant growth factors, and more recently stem cell and exosome-based strategies—truly predictable, complete regeneration of the three-tissue periodontal complex remains elusive [3,4]. The limitations of periodontal regeneration are multifactorial, reflecting defect morphology, wound stability, vascularisation, microbial control, progenitor-cell availability, and patient-related factors; among these, the chronic inflammatory microenvironment of the periodontal pocket is a major, though not the sole, contributor, suppressing developmental pathways that would otherwise support tissue formation.

Uterine Sensitization-Associated Gene-1 (USAG-1), also designated SOSTDC1 (Sclerostin Domain-Containing Protein 1), ectodin, or WISE, has recently attracted extraordinary clinical attention following the entry of an anti-USAG-1 monoclonal antibody (TRG-035, Toregem BioPharma) into first-in-human (Phase 1) testing, reportedly initiated in late 2024, with congenital tooth agenesis as the lead indication. Sponsor announcements indicate that this trial has been completed and that a phase IIa study is being prepared, but we did not identify a peer-reviewed report of the clinical results at the time of writing; the peer-reviewed preclinical antibody-development studies underpinning this programme are therefore cited directly below [5,6]. USAG-1 is a secreted 28 kDa protein that acts as a dual antagonist of BMP and Wnt signalling—the two most fundamental morphogenetic cascades governing tooth development and dental tissue homeostasis [7,8]. Its inhibition in mouse and ferret models produces supernumerary teeth by reactivating dormant odontogenic programmes, indicating that in these models, a primary role of USAG-1 is to restrain tooth formation [5,7].

What has received far less attention is the observation that USAG-1 has been reported to be expressed not only in developing dental tissues but also in gingival fibroblasts, basal gingival epithelial cells, and periodontal tissues in the adult, on the basis of transcript-level data [9]. BMP and Wnt signalling are indispensable for cementum formation, PDL fibre orientation, alveolar bone homeostasis, and the osteogenic differentiation of periodontal ligament stem cells (PDLSCs)—the progenitor population responsible for periodontal regeneration [10,11]. This convergence raises a clinically important question that has not been formally addressed in the literature: could localised USAG-1 inhibition amplify the endogenous BMP/Wnt signalling that drives periodontal tissue regeneration, and if so, through what mechanisms?

This perspective argues that USAG-1 represents a biologically plausible, hypothesis-generating candidate target for the next generation of periodontal regenerative therapy, whose periodontal relevance has not yet been directly tested. We set out the molecular rationale, map the plausible therapeutic pathways, address the challenges that would need to be overcome, and propose a research agenda for testing this hypothesis.

### Evidence Hierarchy and Literature Basis

To make the evidentiary status of each claim in this perspective transparent, we distinguish the following throughout this manuscript: (i) experimentally established USAG-1 biology (largely from developmental and renal contexts); (ii) established periodontal BMP/Wnt biology (largely from non-USAG-1 studies); (iii) indirect evidence bearing on a periodontal role for USAG-1; and (iv) entirely hypothetical therapeutic applications. Table 1 summarizes this evidence hierarchy for the central propositions underlying the hypothesis. The literature underlying this perspective was identified through targeted searches through PubMed/MEDLINE and Google Scholar (records to 2026) using the terms USAG-1, SOSTDC1, BMP antagonism, canonical/non-canonical Wnt signalling, periodontal regeneration, and periodontal ligament stem cells, prioritising peer-reviewed primary research over secondary or promotional sources; this is a narrative perspective and not a systematic review.

## 2. USAG-1 Biology: Structure, Expression, and Signalling

### 2.1. Molecular Identity and Signalling Mechanism

USAG-1 is a member of the cystine-knot superfamily that contains a DAN (Differential screening-selected gene Aberrant in Neuroblastoma) domain with homology to sclerostin. It exerts its inhibitory effects through two distinct binding mechanisms [7,8]. First, it binds directly to BMP-2, BMP-4, BMP-7, and BMP-10, preventing their interaction with type I and type II BMP receptors and thereby suppressing SMAD1/5/8 phosphorylation and downstream transcription of osteogenic and cementogenic genes [7]. Second, it binds to the Wnt co-receptors LRP5 and LRP6, blocking canonical β-catenin signalling [7,17]. In tooth development, the functional consequence of simultaneous BMP and Wnt suppression is arrest of tooth bud progression; that an analogous suppression of cementoblast differentiation and periodontal progenitor activation occurs in the adult periodontium has been proposed but not demonstrated. USAG-1 knockout produces supernumerary tooth formation via progressive rescue of rudimentary tooth buds, consistent with a physiological role in enforcing a developmental ceiling rather than merely modulating signalling intensity [7,8], as shown in Figure 1.

### 2.2. Expression in Periodontal Tissues

A recent peer-reviewed literature review re-analysing public single-cell RNA sequencing databases reports that USAG-1 expression extends to adult human gingival tissue beyond the developing tooth [9], identifying USAG-1 transcripts in distinct subsets of human gingival fibroblasts (HGFs; CD9^+^APCDD1^+^ and CDH19^+^LAMA2^+^ populations) and in a subset of Krt14^+^ basal gingival epithelial cells. This source is a peer-reviewed re-analysis of public transcriptomic data rather than a study specifically designed to quantify periodontal expression, and it does not report on periodontitis-affected tissue. Whether USAG-1 protein is secreted and functionally available at the periodontal interface in vivo and whether its expression changes in periodontitis remain open empirical questions (Table 1; Section 6). This cell-specific distribution is potentially significant: the gingival epithelial–mesenchymal interface is the anatomical site at which epithelial–mesenchymal interactions fundamental to periodontal tissue induction occur [18]. Furthermore, USAG-1 has been characterised as a BMP antagonist abundantly expressed in the kidney [19] and has also been reported in gingival and dental tissues [9].

In the context of periodontitis, the inflammatory microenvironment modulates both BMP and Wnt signalling. Dkk-1, another Wnt antagonist, is upregulated in periodontitis and contributes to alveolar bone loss by suppressing Wnt-driven osteogenesis [16]. Deletion of Dkk-1 in osteocytes reduced experimental alveolar bone loss by 28.5% in a murine ligature-induced periodontitis model, indicating that osteocytic Dkk-1 contributed to bone loss in this model; the extent to which Wnt antagonism drives bone destruction in human periodontitis remains to be established [16]. USAG-1’s dual antagonism of both BMP and Wnt pathways suggests it may function as a compounding suppressor in the inflamed periodontium, concurrently blocking two independent regenerative signals at a site where both are needed.

## 3. BMP and Wnt Signalling in Periodontal Tissue Biology

### 3.1. Role of BMP Signalling in the Periodontium

Bone morphogenetic proteins are among the most potent inducers of periodontal tissue regeneration identified to date. BMP-2, BMP-7, and BMP-12 have each been shown to promote alveolar bone regeneration, cementum formation, and PDL fibre insertion in animal models [20,21]. Recombinant human BMP-2 (rhBMP-2) delivered on collagen scaffolds produces significant alveolar bone and PDL regeneration in canine critical-size defect models [22]. BMP-7 is particularly relevant to cementogenesis: it promotes the differentiation of PDL fibroblasts into cementoblast-like cells expressing F-spondin and other cementogenic markers through the canonical BMP-SMAD pathway and cementoblasts express and secrete BMP-7 in an autocrine regulatory loop [12].

BMP signalling also regulates the lineage commitment of PDLSCs. In human PDLSCs in vitro, the effect of BMP-4 on lineage bias is dose-dependent: low-dose BMP-4 (10 ng/mL) sustains multipotency, upregulating both the osteogenic marker RUNX2 and the tenogenic (ligament-associated) marker SCX, whereas high-dose BMP-4 (100 ng/mL) favours tenogenic differentiation and suppressed osteogenic commitment [13] (Figure 2). PDL cell fate in culture is likewise dependent on the growth factor applied: BMP-7 favours a mineralised, cementoblast-like fate, whereas other factors favour a non-mineralised fibroblastic fate or maintenance of a naiver phenotype [23]. These findings caution that the direction of any change in PDLSC differentiation cannot be inferred simply from increased BMP signalling. If USAG-1 does suppress BMP signalling tonically in the adult periodontium—a premise that has not been directly demonstrated (Table 1)—its inhibition might shift PDLSC differentiation toward mineralised tissue formation or toward ligament-like differentiation, or it may have no net regenerative effect, depending on the level of BMP activity achieved, the receptor context, and the inflammatory milieu. Any USAG-1-mediated modulation of PDLSC differentiation is therefore a hypothesis rather than an established mechanism.

### 3.2. Role of Wnt Signalling in the Periodontium

In vitro studies indicate that canonical Wnt/β-catenin signalling can support the viability and osteogenic differentiation of PDLSCs and the osteogenic capacity of gingival fibroblasts, although these data are derived largely from in vitro systems and are in part preliminary [14,15]. PDL cells stimulate osteogenic capacity in gingival fibroblasts through paracrine Wnt signalling, an effect that was only partially inhibited by the Wnt antagonist Dkk-1 [14]. Wnt5a, a non-canonical Wnt ligand, is upregulated in LPS-stimulated PDLSCs as part of an NF-κB-mediated inflammatory cascade, suggesting that Wnt pathway dysregulation may contribute to the impaired regenerative potential of PDLSCs in an inflammatory environment (an in vitro observation) [24], as shown in Figure 3.

In an in vitro study, gingival stem cell-conditioned media, both alone and combined with low-level laser therapy, enhanced Wnt and TGF-β signalling components at the transcript level in PDLSCs together with RUNX2, ALP, and osteocalcin expression and mineralisation [15]. The authors of that study regarded the pathway data as preliminary. Taken together, these largely in vitro observations support the view that Wnt signalling is relevant to periodontal regeneration, but its mechanistic importance in vivo remains to be established. USAG-1, by blocking LRP5/6-mediated Wnt co-receptor activation, is hypothesised to suppress this regenerative axis, although this has not been directly tested in periodontal tissue (Figure 4).

## 4. Hypothesised Mechanisms by Which USAG-1 Inhibition Could Influence Periodontal Regeneration

Based on the foregoing biology, we identify five hypothesised mechanisms, each currently supported only indirectly (Table 1), through which local USAG-1 inhibition could plausibly enhance periodontal tissue regeneration.

### 4.1. Amplification of BMP-Driven Cementogenesis

Cementum is the tissue most resistant to regeneration in the periodontium. Its formation requires sustained BMP signalling to drive dental follicle cell and PDLSC differentiation into cementoblasts expressing cementum attachment protein, cementum protein-1, and alkaline phosphatase [12,25]. If USAG-1 restrains BMP signalling at the adult root surface—which has not been demonstrated—its inhibition might partially recapitulate aspects of the BMP signalling environment present during root development, when Hertwig’s epithelial root sheath (HERS) disintegration exposes dental follicle cells to BMP signals that initiate cementogenesis [25]. This putative mechanism might be particularly attractive for the treatment of infrabony defects, where the root surface is already exposed and accessible for local drug delivery, and where the residual PDLSC population may retain cementogenic potential, an assumption that requires testing.

### 4.2. Reactivation of PDLSC Osteogenic and Fibrogenic Differentiation

PDLSCs express BMP receptors and respond to BMP signalling in vitro with osteogenic and tenogenic (ligament-like) differentiation [13], and BMP-7 has been reported to promote a cementoblast-like phenotype in PDL fibroblasts [12]. It has been proposed, but not demonstrated, that endogenous USAG-1 produced by adjacent gingival fibroblasts restrains BMP signalling in the chronically inflamed periodontium and thereby limits PDLSC differentiation. If so, local USAG-1 inhibition at the time of regenerative surgery might alter the PDLSC differentiation programme, possibly toward mineralised tissue formation, without the supraphysiological BMP doses that have been associated with ankylosis and root resorption after exogenous rhBMP-2 delivery [20,22]. However, given the dose-dependent lineage effects noted in Section 3.1, neither the direction nor the magnitude of any shift in PDLSC differentiation can be predicted from the current evidence. Relief of Wnt suppression might additionally support PDLSC viability or proliferation, potentially enlarging the progenitor pool—an additive effect that remains to be demonstrated rather than assumed [15].

### 4.3. Amplification of Wnt-Driven Alveolar Bone Repair

The alveolar bone defect—vertical or horizontal—is the structural consequence of periodontitis that most limits tooth retention and implant placement [1,2]. Wnt signalling through LRP5/6 is thought to promote osteoblast proliferation and bone formation, and its suppression by Dkk-1 is implicated in alveolar bone loss in experimental periodontitis [16]. USAG-1 inhibits the same LRP5/6 co-receptor. Local anti-USAG-1 delivery to the osseous defect, potentially within a bone graft carrier, could relieve Wnt antagonism at the osteoblast–bone interface simultaneously with BMP upregulation, potentially producing an additive or synergistic pro-osteogenic microenvironment—a combination effect that has not been tested for USAG-1 specifically. In a murine ligature-induced periodontitis model, osteocyte-specific Dkk-1 deletion produced not only reduced bone loss but increased bone mineral density and trabecular thickness in inflamed alveoli [16]. The magnitude of this effect cannot presently be assumed for anti-USAG-1 therapy: USAG-1 and Dkk-1 are not interchangeable molecular targets, and the Dkk-1 data are presented here only as proof-of-principle that pharmacological modulation of a Wnt antagonist can influence alveolar bone biology, not as a quantitative prediction for USAG-1 blockade.

### 4.4. Gingival Niche Activation and Epithelial–Mesenchymal Signalling

USAG-1 transcripts have been reported in gingival fibroblast subsets and in basal gingival epithelial cells (transcript-level data from non-diseased tissue) [9]. These cell populations occupy the gingival niche that provides paracrine support to the PDL. Local USAG-1 inhibition in the junctional and sulcular epithelium–fibroblast interface might modulate the epithelial–mesenchymal BMP/Wnt cross-talk that is proposed to be disrupted by periodontitis, with possible, entirely speculative, effects on junctional epithelial re-attachment and on the regenerative gingival niche [18]. This putative mechanism would be distinct from, and potentially complementary to, the direct cementogenic and osteogenic pathways described above.

### 4.5. Potential Compatibility with Existing Regenerative Approaches

Anti-USAG-1 therapy would not replace existing regenerative strategies but might, in principle, complement them; the combinations below are speculative, testable hypotheses rather than established synergistic interactions, and no study has examined USAG-1 inhibition in combination with any of these approaches. In guided tissue regeneration (GTR) with collagen membranes, the membrane is intended to exclude epithelial downgrowth and maintain space for progenitor-cell repopulation; whether USAG-1 inhibition delivered within the membrane space could augment BMP- and Wnt-dependent progenitor differentiation, and whether any such augmentation would be beneficial, is unknown [3,4]. Enamel matrix derivative (Emdogain^®^) is thought to influence several signalling pathways, and its precise mechanism of action remains incompletely defined; whether USAG-1 inhibition would enhance, or be redundant with, its clinical effect in deep defects is untested and, at present, speculative [4]. With bone graft materials such as Bio-Oss^®^, which provide an osteoconductive scaffold, USAG-1 inhibition could in principle add an osteoinductive BMP/Wnt stimulus that xenografts alone may lack, but this remains an untested hypothesis rather than an established mechanism, and any reduction in dependence on endogenous growth-factor adsorption would be speculative.

## 5. Proposed Delivery Strategies

The systemic administration model used in the current TRG-035 clinical trials—intravenous antibody delivery targeting tooth agenesis—is unlikely to be appropriate for a periodontal regenerative application; targeted local delivery would be preferable in order to limit off-target effects on bone remodelling elsewhere and on renal BMP signalling given the renal expression of USAG-1 [19]. We propose four localised delivery approaches that warrant investigation. Among these, local antibody delivery (via collagen membrane or graft carrier, described first below) is prioritised here as the most conceptually straightforward approach because an antibody already exists and collagen membranes and grafts are in routine clinical use, although its feasibility for periodontal application remains untested; the nucleic-acid- and small-molecule-based platforms described later in this section remain longer-term, more speculative possibilities that require substantially more upstream validation before they could be considered translationally feasible.

Collagen membrane-incorporated antibody: The anti-USAG-1 antibody could, in principle, be incorporated into the collagen matrix of a resorbable GTR membrane to provide local release during the early phase of wound healing. Collagen membranes are already used clinically for GTR, but we are not aware of studies evaluating them as carriers for this or comparable antibodies in periodontal defects; release kinetics, antibody integrity, and retention of neutralising activity within cross-linked collagen matrices would need to be demonstrated experimentally, and the duration of local exposure required for any regenerative effect is unknown.

Antibody-functionalised bone graft carrier: Anti-USAG-1 antibody loaded onto a granular xenograft carrier (conceptually analogous to the rhBMP-2/absorbable collagen sponge system) might concentrate the active agent within the osseous defect while providing an osteoconductive scaffold. Whether a mineralised xenograft can retain and release an intact, active antibody at useful rates is unknown and would need to be established experimentally. This concept parallels preclinical experience with growth-factor-loaded carriers [22], which does not in itself demonstrate feasibility for antibodies.

Gene therapy or siRNA-mediated local silencing: Local gene-silencing approaches—for example, adeno-associated viral (AAV) vectors encoding USAG-1 shRNA, CRISPR-mediated silencing in PDLSCs, or local siRNA—could in principle provide more sustained USAG-1 suppression without repeated antibody dosing, but each raises additional safety and regulatory questions (including vector immunogenicity, off-target effects, and the difficulty of reversing a sustained effect) that would be particularly important given the Wnt-related considerations discussed in Section 6. The only local Usag-1 siRNA data of which we are aware come from tooth-development models, in which topically applied Usag-1 siRNA, with cationised gelatin as a delivery system, partially rescued arrested tooth development in Runx2-deficient mice [26]. We are not aware of any application of Usag-1 siRNA, nanoparticle-based or otherwise, to periodontal tissues; this remains an early-stage, longer-term possibility rather than a near-term, translationally feasible platform for periodontal delivery.

USAG-1-blocking peptides or small-molecule inhibitors: A structure-based virtual-screening study, so far available only as a non-peer-reviewed preprint, proposed candidate small-molecule USAG-1 ligands in silico; these remain at a purely computational stage, with no experimental validation of target engagement or efficacy reported to date, and would need substantial preclinical development before formulation as sustained-release microspheres for intra-pocket delivery could be considered [27]. If validated experimentally, small-molecule inhibitors could eventually offer advantages over antibodies in manufacturing simplicity, thermal stability, and cost of production—important considerations for equitable clinical deployment, but currently a longer-term prospect.

## 6. Challenges and Limitations

Off-target BMP suppression and renal safety: USAG-1 plays physiologically important roles in renal BMP signalling; systemic USAG-1 inhibition could disrupt normal kidney function. Given this physiological role, local rather than systemic delivery is not merely a formulation preference but a safety-critical design requirement for any periodontal application [19]. Localised delivery strategies are therefore strongly preferred, and likely necessary, pending pharmacokinetic and safety data. All preclinical models must include renal histopathology and renal BMP pathway markers as safety endpoints.

Context dependency of BMP signalling in periodontitis: The chronic inflammatory microenvironment of the periodontal pocket suppresses downstream BMP signalling at multiple levels beyond USAG-1. TNF-α, IL-1β, and bacterial LPS each inhibit SMAD signalling, and pro-inflammatory cytokines drive RANKL expression that overwhelms any anabolic BMP/Wnt stimulus [28]. USAG-1 inhibition must therefore be applied after adequate mechanical debridement and inflammatory suppression—embedded within the EFP stepwise algorithm, not as a substitute for it. If inflammation is inadequately resolved before any anti-USAG-1 intervention, the resulting inflammatory signalling could neutralise the intended anabolic effect entirely, independent of whether USAG-1 blockade itself is effective.

Risk of root resorption and ankylosis: Supraphysiological BMP stimulation at the root surface can cause root resorption and ankylosis, as documented with high-dose rhBMP-2 applications [20]. USAG-1 inhibition, because it amplifies endogenous BMP rather than delivering exogenous supraphysiological doses, may avoid this complication—but this must be verified in root-proximity models with detailed histomorphometric analysis of cementum–root surface interfaces.

Wnt pathway complexity and cancer risk: Aberrant activation of Wnt/β-catenin signalling is associated with carcinogenesis in several tissues [29], and increased β-catenin activity has been reported in oral epithelial dysplasia and oral squamous cell carcinoma [30]. These associations reflect, depending on the tissue, oncogenic pathway mutations or sustained ligand overproduction, and do not by themselves show that transient, local relief of Wnt antagonism would be carcinogenic; nevertheless, they warrant caution for any strategy intended to enhance Wnt signalling near the oral epithelium. Non-canonical Wnt5a upregulation has also been linked to inflammatory responses in PDLSCs in vitro [24]. In addition, USAG-1/Wise has been reported to act as both an activator and an inhibitor of Wnt signalling depending on context (in Xenopus assays) [17], so the net effect of its inhibition on Wnt activity in the periodontium cannot be assumed. A related but distinct concern is the theoretical long-term risk that repeated or sustained local Wnt/β-catenin reactivation could promote oral epithelial proliferation or, in principle, dysplastic change; no data address this for USAG-1 inhibition, and it should be monitored explicitly as a separate safety endpoint. Long-term safety monitoring for oral epithelial dysplasia must accompany any sustained USAG-1 inhibition strategy, and the dose–response relationship between Wnt reactivation and cell proliferation must be characterised.

Additional local risks: Beyond root resorption, ankylosis, and epithelial dysplasia, local USAG-1 inhibition could plausibly also produce ectopic mineralisation outside the intended defect, altered bone remodelling at sites adjacent to the delivery zone, interference with normal physiological root remodelling, immune responses to repeated local antibody administration, and effects on neighbouring dental tissues or on the eruption of unerupted or developing teeth in younger patients. None of these has been directly assessed for USAG-1 blockade in the periodontium and each should be an explicit safety endpoint in preclinical work.

Plausible negative or null outcomes: A rigorous evaluation of this hypothesis must also consider outcomes that would argue against it. USAG-1 expression could conceivably decrease, rather than increase, in periodontitis, which would undermine the rationale for local inhibition. USAG-1 blockade might enhance bone formation while impairing PDL fibre organisation, yielding ankylosis rather than functional regeneration. Wnt reactivation might preferentially favour epithelial proliferation over mesenchymal regeneration. Finally, it is possible that cementogenesis and ankylosis cannot be experimentally separated with current histological methods, which would complicate interpretation of any efficacy signal. These possibilities should be built into the research agenda below as explicit falsification criteria, not treated only as risks to be managed after a positive result.

Absence of direct evidence: At present, no study has directly examined USAG-1 expression in periodontitis versus healthy periodontal tissues, nor has any study tested the effect of USAG-1 inhibition in an animal model of periodontitis with regenerative endpoints. This perspective is therefore mechanistic and inferential, grounded in the convergence of established USAG-1 biology and periodontal signalling science, but requiring direct experimental validation.

## 7. Proposed Research Agenda

The following research steps are required to translate this hypothesis into clinical evidence:

- Step 1—Characterise USAG-1 expression in human periodontal health and disease: This step, and specifically immunohistochemical (protein-level) profiling of USAG-1 in periodontitis-affected versus healthy tissue, is the necessary first priority and should precede all downstream in vitro and animal work, since it establishes baseline pathological relevance for the entire hypothesis. Study immunohistochemistry and single-cell RNA sequencing of paired periodontal tissue biopsies from healthy controls and Stage II and Stage III/IV periodontitis patients to quantify USAG-1 expression in gingival fibroblasts, basal epithelial cells, PDL, cementoblasts, and alveolar bone osteocytes. Correlate USAG-1 levels with BMP-2/4/7 and Wnt3a tissue concentrations.
- Step 2—In vitro USAG-1 inhibition in PDLSCs under inflammatory conditions: Treat PDLSCs with anti-USAG-1 antibody or siRNA under LPS-stimulated inflammatory conditions. Assess osteogenic/cementogenic differentiation (RUNX2, ALP, CAP, CEMP-1), mineralisation assays, and proliferation relative to untreated and rhBMP-2 controls.
- Step 3—Ligature-induced periodontitis animal model with local anti-USAG-1 delivery: Establish experimental periodontitis in a rodent or beagle model. At the time of definitive flap surgery, apply localised anti-USAG-1 antibody in a collagen membrane carrier, with a full comparator design comprising a defect/periodontitis-only control, standard regenerative therapy alone, anti-USAG-1 alone, standard therapy combined with anti-USAG-1, and a vehicle (carrier-only) control. Endpoints: Micro-CT alveolar bone volume, histomorphometric cementum area, PDL fibre orientation, and root surface ankylosis assessment, together with connective tissue attachment level, epithelial downgrowth, and root resorption, at 8 and 16 weeks.
- Step 4—Combination efficacy studies: Test anti-USAG-1 combined with Emdogain^®^, Bio-Oss^®^, and PRF (platelet-rich fibrin) in the animal model to identify the optimal combination for clinical translation.
- Step 5—Safety profiling: Assess renal histopathology, serum BMP markers, systemic bone density assessment, and oral epithelial proliferation indices across all animal dosing experiments.
- Step 6—Long-term translational outlook (first-in-human trial, contingent on Steps 1–5): A first-in-human study is premature until a minimum translational evidence package is assembled, comprising confirmed periodontal target expression, evidence of pathway engagement, dose–response data, local and systemic biodistribution, toxicology, assessment of tumorigenic potential, and reproducible regenerative efficacy—including absence of unacceptable root resorption or ankylosis—in an appropriate animal model. Only once these are satisfied would a Phase 1 periodontal trial, testing safety and preliminary efficacy in Stage III infrabony defects with local collagen-carrier anti-USAG-1 delivery following complete non-surgical therapy and resolution of acute inflammation, become appropriate. Primary endpoint: Safety. Secondary endpoints: Radiographic bone gain, clinical attachment gain, and probing depth reduction at 12 months.

## 8. Integration with the Existing Periodontal Treatment Framework

The European Federation of Periodontology S3-level guideline provides a stepwise treatment algorithm in which regenerative surgery occupies Step 3, applied to residual infrabony defects and furcation involvements after adequate Step 1 and Step 2 mechanical therapy [3]. Anti-USAG-1 therapy, if validated, would be positioned at Step 3 as a biological adjunct to access surgery—not a replacement for it. The preconditions are the same as for current regenerative protocols: controlled inflammation, adequate plaque control, non-smoking status, and stable systemic disease. The rationale for placing it at Step 3 rather than earlier in the algorithm is that the inflammatory microenvironment at Steps 1 and 2 would overwhelm any anabolic BMP/Wnt stimulus, as outlined in Section 6.

If USAG-1 inhibition is eventually validated, illustrative future candidate phenotypes might include Stage III infrabony defects, furcation class II defects in accessible locations, and selected Stage IV cases in the context of full-arch rehabilitation—the patients in whom current regenerative options are least predictable. These specific thresholds are illustrative only and are not derived from any USAG-1-specific evidence; precise patient-selection criteria would need to be established empirically once efficacy data exist, not proposed in advance of them.

## 9. Conclusions

USAG-1 is more than a brake on tooth formation. It has been reported to be expressed in gingival and periodontal tissues of the adult and, on the basis of its dual antagonism of BMP and Wnt signalling in other contexts, is hypothesised to modulate the same molecular machinery implicated in cementogenesis, PDL fibre formation, and alveolar bone repair—though this periodontal role has not yet been directly demonstrated. The convergence of established USAG-1 biology with what is known about periodontal tissue regeneration creates a mechanistically plausible, testable case for its therapeutic inhibition in the context of regenerative periodontal surgery, rather than a demonstrated one.

Three theoretical features would, if confirmed, distinguish this approach from current regenerative options: endogenous amplification of the body’s own signalling rather than delivery of exogenous growth factors at supraphysiological doses; simultaneous engagement of two independent regenerative axes (BMP and Wnt) rather than one; and a cell-specific gingival expression profile that could in principle allow more targeted biological activity. None of these three features has yet been demonstrated to translate into a periodontal regenerative benefit, and USAG-1 is best regarded, at this stage, as a candidate regulatory target whose periodontal function remains experimentally undefined, rather than as a validated therapeutic target.

The evidence presented here is inferential, constructed from the convergence of established molecular biology with clinical periodontal science. Direct experimental evidence in periodontal tissues is absent, and generating it is the first obligation of this research agenda. The current clinical momentum of USAG-1 inhibition in tooth regeneration may provide scientific infrastructure, antibody tools and, in time, regulatory experience on which a periodontal regenerative programme could potentially be built efficiently. Whether USAG-1 will join enamel matrix derivatives and recombinant BMP-2 as a validated periodontal regenerative agent depends on whether the field will invest in testing this hypothesis directly.

## Figures and Tables

**Figure 1:**
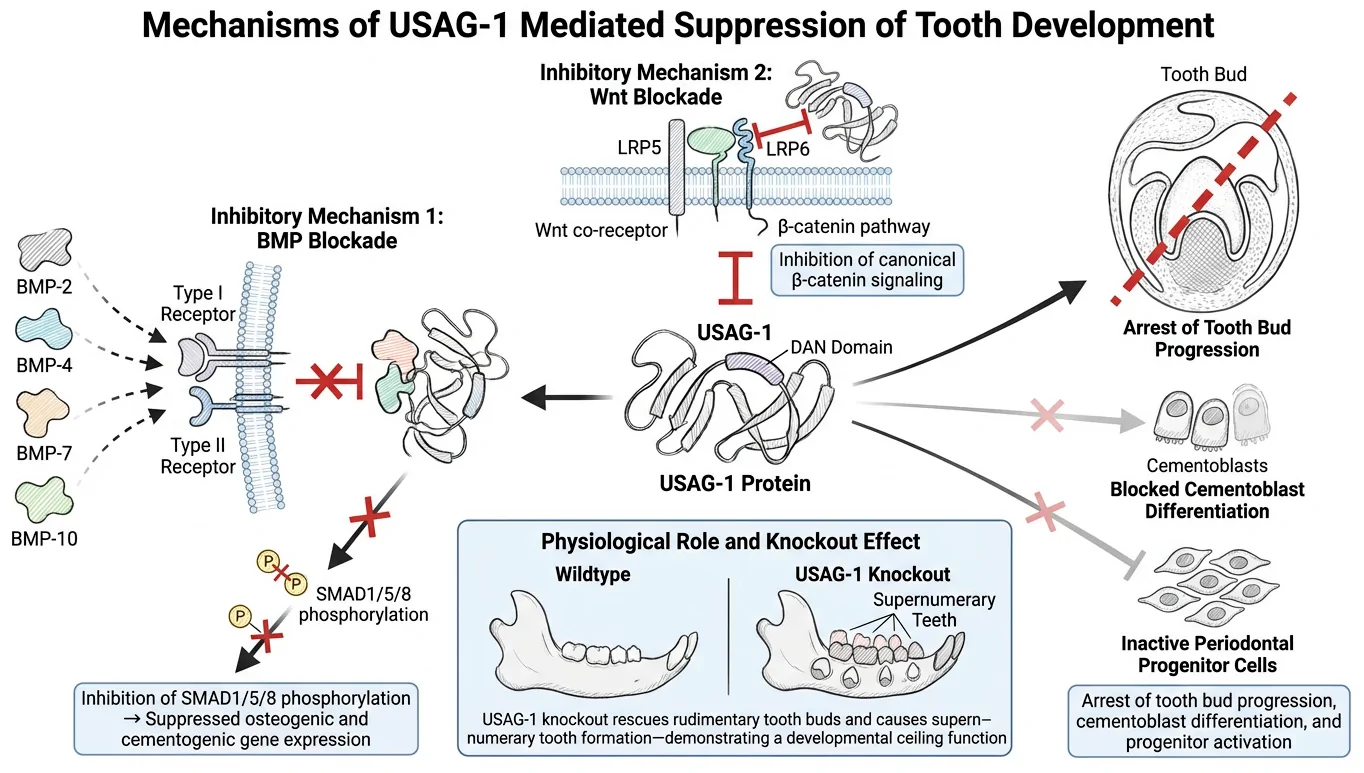
Schematic illustration of the mechanisms by which USAG-1 suppresses tooth development. USAG-1 inhibits odontogenesis through two major pathways: suppression of BMP signalling via interference with BMP receptor activation and inhibition of Wnt/β-catenin signalling through LRP5/6 blockade. These inhibitory effects arrest tooth bud progression; a corresponding impairment of cementoblast and periodontal progenitor differentiation is hypothesised but has not been demonstrated. Conversely, USAG-1 knockout promotes rescue of rudimentary tooth buds and the formation of supernumerary teeth. Red flat-ended (T-bar) lines indicate inhibition by USAG-1 of BMP receptor binding, LRP5/6 co-receptor activation, and canonical β-catenin signalling; the red cross indicates blocked SMAD1/5/8 phosphorylation (P, phosphate group); the solid black arrow and the red dashed line across the tooth bud indicate the established outcome, arrest of tooth bud progression; and the lighter grey arrows marked with a cross indicate the hypothesised, not yet demonstrated, suppression of cementoblast differentiation and periodontal progenitor activation. Created with FigureLab v2.1.

**Figure 2:**
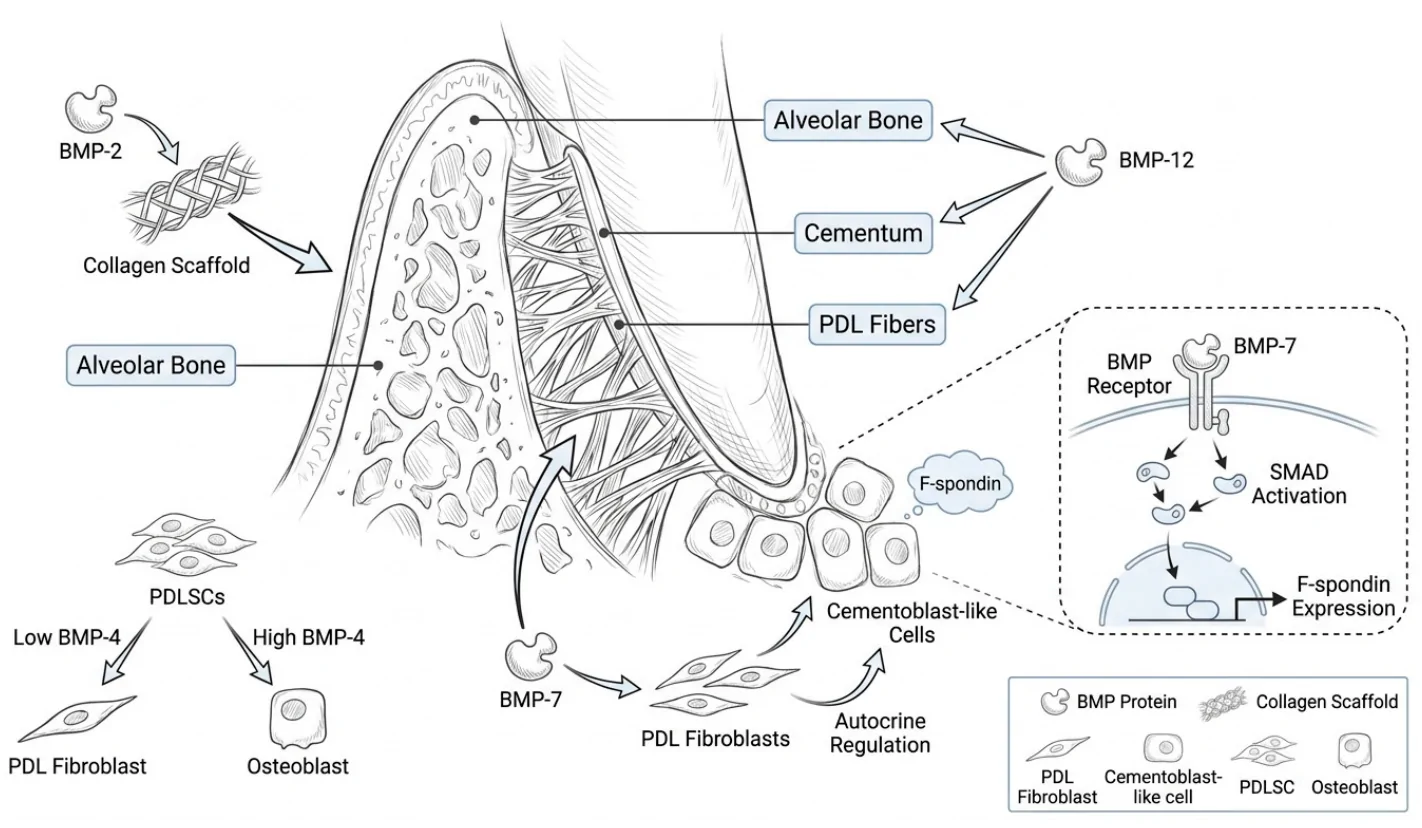
Schematic representation of BMP-mediated periodontal and cementum regeneration. BMP-2, BMP-4, BMP-7, and BMP-12 promote differentiation of periodontal ligament stem cells (PDLSCs) into osteoblasts, PDL fibroblasts, and cementoblast-like cells through BMP receptor-mediated SMAD activation. BMP-7 enhances expression of F-spondin and supports autocrine regulation of cementogenesis. These coordinated signalling pathways contribute to alveolar bone formation, periodontal ligament organisation, and cementum regeneration. Arrows indicate the actions of individual BMPs on their target tissues or cells and the direction of cell differentiation; the dashed inset shows an enlarged view of BMP-7 binding to its receptor, SMAD activation, and F-spondin expression in cementoblast-like cells. Cell types and materials are identified in the key. Created with FigureLab v2.1.

**Figure 3:**
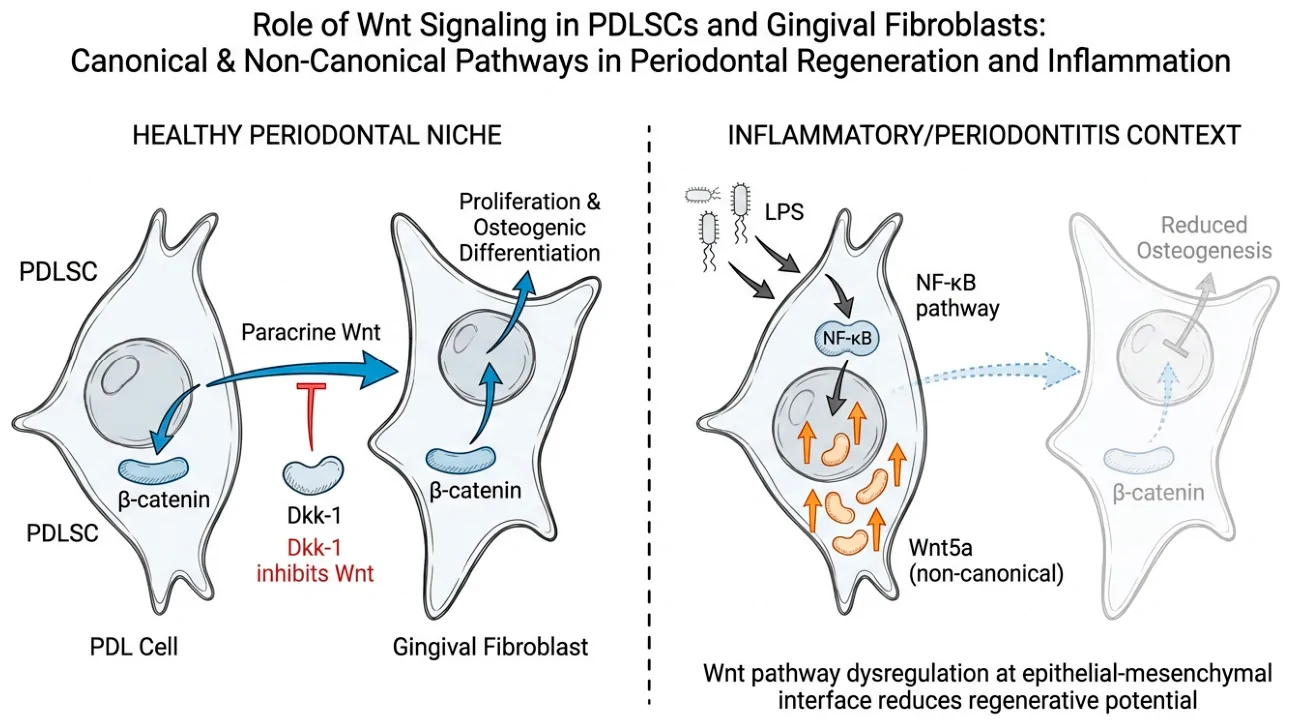
Schematic illustration of canonical and non-canonical Wnt signalling pathways in periodontal ligament stem cells (PDLSCs) and gingival fibroblasts under healthy and inflammatory conditions. In healthy periodontal tissues, canonical Wnt/β-catenin signalling promotes cellular proliferation, osteogenic differentiation, and tissue regeneration, while inhibitors such as Dkk-1 modulate pathway activity. Under inflammatory or periodontitis conditions, dysregulated Wnt signalling and NF-κB activation reduce osteogenic potential and impair periodontal regenerative capacity. Blue arrows indicate canonical (paracrine) Wnt/β-catenin signalling and its downstream effects; the red flat-ended (T-bar) line indicates Dkk-1-mediated inhibition of Wnt signalling; grey arrows indicate LPS stimulation leading to NF-κB pathway activation; orange arrows indicate inflammation-associated non-canonical (Wnt5a) signalling; and the faded cell with the dashed, translucent arrow indicates reduced osteogenic capacity under inflammatory conditions. Created with FigureLab v2.1.

**Figure 4:**
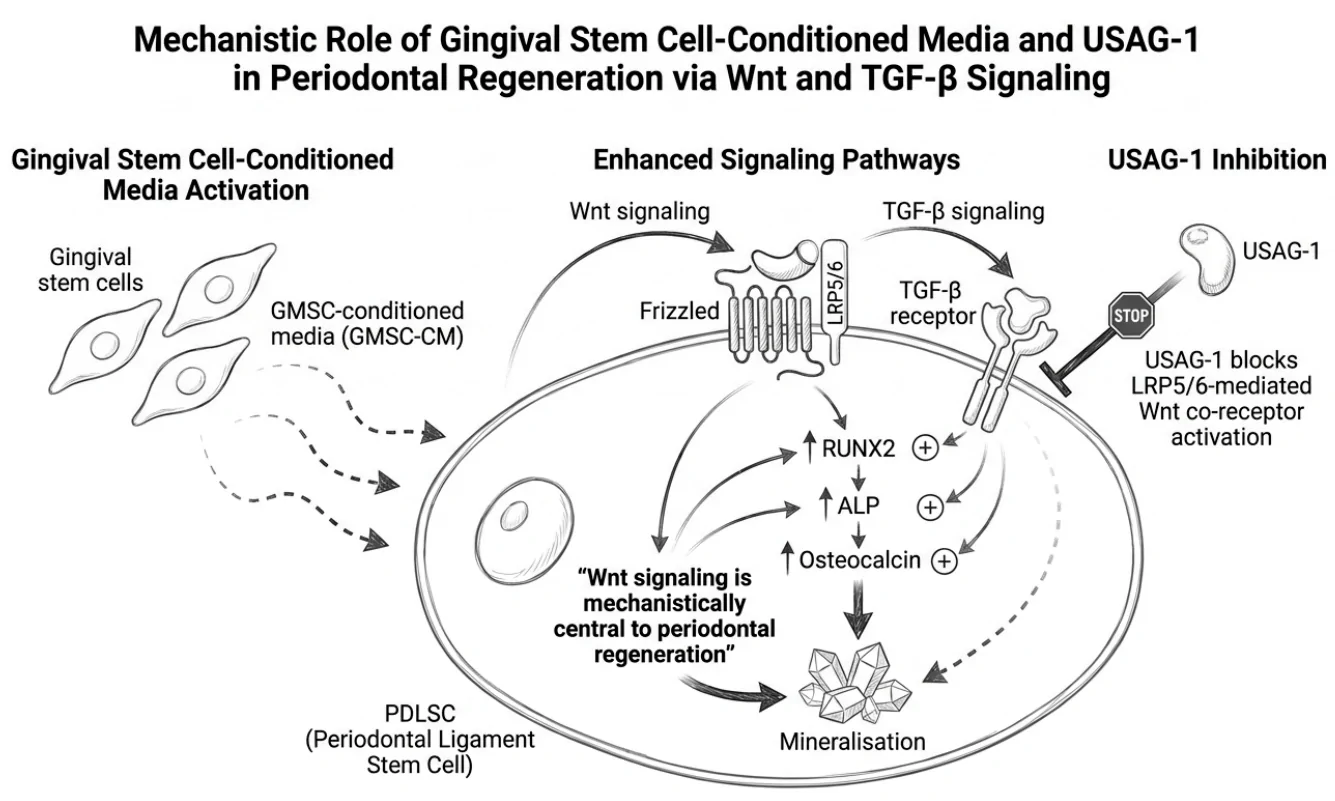
Schematic illustration of gingival stem-cell-conditioned medium enhancing Wnt and TGF-β signalling components in periodontal ligament stem cells (PDLSCs), and of the hypothesised effect of local USAG-1 inhibition on this axis. Conditioned medium upregulates RUNX2, ALP, and osteocalcin expression and promotes mineralisation in vitro; USAG-1 blockade is hypothesised, but not yet experimentally shown, to further potentiate this effect by relieving co-receptor (LRP5/6) antagonism. Dashed arrows indicate paracrine delivery of GMSC-conditioned medium (GMSC-CM) to PDLSCs; solid arrows indicate signalling activation and downstream effects; upward arrows (↑) indicate upregulated expression; (+) indicates a stimulatory effect on osteogenic marker expression and mineralisation; the flat-ended (T-bar) line indicates USAG-1-mediated inhibition of LRP5/6 co-receptor activation; and the stop symbol indicates the proposed therapeutic blockade of USAG-1. Created with FigureLab v2.1.

**Table 1:** Evidence versus hypothesis: The central molecular propositions underlying this perspective, graded by evidentiary status.

Molecular Proposition	Evidence Source	Experimental System	Direct Periodontal Evidence?	Level of Certainty	Critical Experiment Required
USAG-1 is a dual BMP/Wnt antagonist	Murashima-Suginami 2008; Laurikkala 2003 [7,8]	Mouse knockout; biochemical binding assays	No (non-periodontal tissue)	Established (developmental biology)	Not required—already established
USAG-1 is expressed in adult human gingival fibroblasts and basal epithelium	Moradi et al., 2026 [9]	Public single-cell RNA-seq database re-analysis	Partial (gingiva; not diseased periodontium specifically)	Indirect/preliminary	IHC/scRNA-seq of periodontitis vs. healthy tissue (Research Agenda Step 1)
BMP signalling drives cementoblast and PDLSC osteogenic differentiation	Iwasawa et al., 2025 [12]; Li et al., 2025 [13]	In vitro PDL cell/PDLSC culture; one rat root canal model	Limited (predominantly in vitro)	Supported (periodontal, in vitro; dose- and context-dependent)	In vivo confirmation with USAG-1 modulation specifically; define dose-dependent lineage effects
Canonical Wnt signalling supports PDLSC/gingival fibroblast proliferation and osteogenesis	Garna et al., 2022 [14]; Aljabri et al., 2025 [15]	In vitro cell culture/paracrine co-culture	No (in vitro only)	Supported (periodontal, in vitro; partly preliminary)	Confirmation of USAG-1-specific contribution vs. other Wnt antagonists
Dkk-1 blockade reduces alveolar bone loss in experimental periodontitis	Goes et al., 2019 [16]	Osteocyte-specific knockout mice	Yes (murine periodontitis)	Established for Dkk-1, NOT for USAG-1	Direct anti-USAG-1 periodontitis animal study (Research Agenda Step 3)
USAG-1 inhibition will enhance periodontal regeneration	(this perspective)	(no direct study exists)	No	Hypothetical	Full research agenda (Steps 1–6 below)

## Data Availability

No new data were created or analyzed in this study. Data sharing is not applicable to this article.
